# Supplementary material for: Structure of the ceramide-bound SPOTS complex
Source: Nat Commun. 2023 Oct 4;14:6196. doi: 10.1038/s41467-023-41747-z (PMC10550967; doi:10.1038/s41467-023-41747-z)
Supplement: Supplementary file 1 — Supplementary Information [file 41467_2023_41747_MOESM1_ESM.pdf]

Supplementary Figures and Tables

Structure of the ceramide-bound SPOTS complex

Jan-Hannes Schäfer[1†], Carolin Körner[2†], Bianca M. Esch[2], Sergej Limar[2], Kristian Parey[1,3], Stefan Walter[3], Dovile Januliene[1,3#], Arne Moeller[1,3#], Florian Fröhlich[2,3#]

[1] Osnabrück University
Department of Biology/Chemistry
Structural Biology section
49076 Osnabrück, Germany

[2] Osnabrück University
Department of Biology/Chemistry
Bioanalytical Chemistry section
49076 Osnabrück, Germany

[3] Osnabrück University
Center of Cellular Nanoanalytic Osnabrück (CellNanOs)
49076 Osnabrück, Germany

[†] These authors contributed equally to this work

[#] For correspondence:
florian.froehlich@uos.de (F. F.); arne.moeller@uos.de (A. M.);
dovile.januliene@uos.de (D. J.)

Keywords: SPOTS complex, Sac1, serine palmitoyltransferase, sphingolipids, ceramide

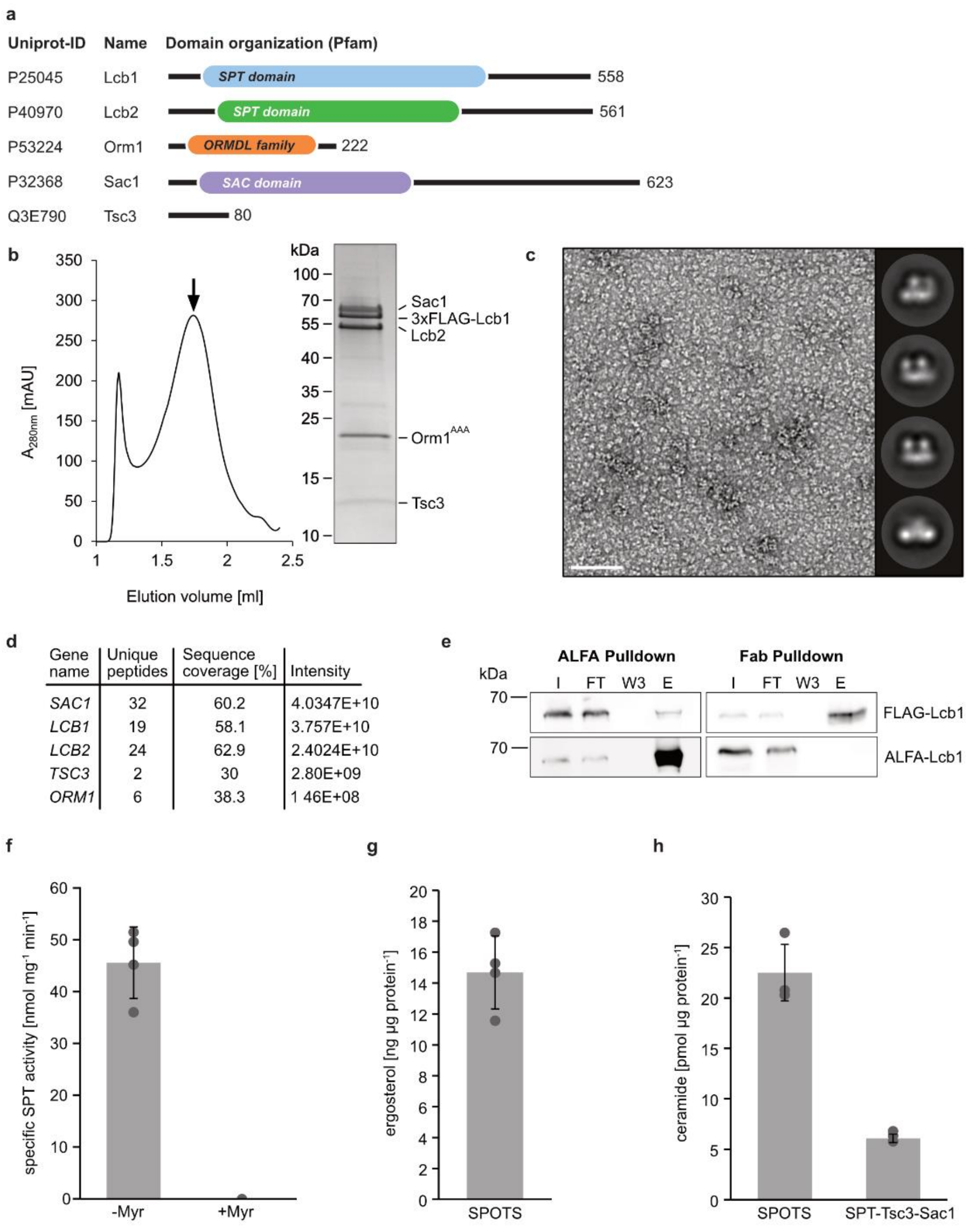

Sup-Fig 1: ***In vitro* functional characterization of the yeast SPOTS complex**

a Pfam-based domain annotation of the SPOTS subunits. **b** SEC profile and corresponding Coomassie-blue stained SDS-PAGE gel of indicated fraction of GDN-solubilized SPOTS complex. **c** Representative micrograph and 2D class averages from negative-stain TEM. 100 nm scale bar. **d** Summary of the mass-spectrometric analysis of the sample in b. **e** ALFA-Lcb1 (left) or FLAG-Lcb1 (right) were pulled down from diploid cells expressing one ALFA-tagged and one FLAG-tagged version of Lcb1. Western blots were probed with antibodies against the ALFA and the FLAG tag. I = input; FT = flow-through; W3 = wash, E = eluate. **f** Specific SPT enzyme activity measurement with or without the specific SPT-inhibitor myriocin (Myr). **g** Ergosterol quantification from GDN-solubilized and purified SPOTS complex. **h** Ceramide quantification from GDN-solubilized and purified SPOTS complex and Orm-free SPT-Tsc3-Sac1 complex. n=4 technically independent samples for f-h and data are presented as mean values ± SD. Source data are provided as a Source Data file for b, e – h.

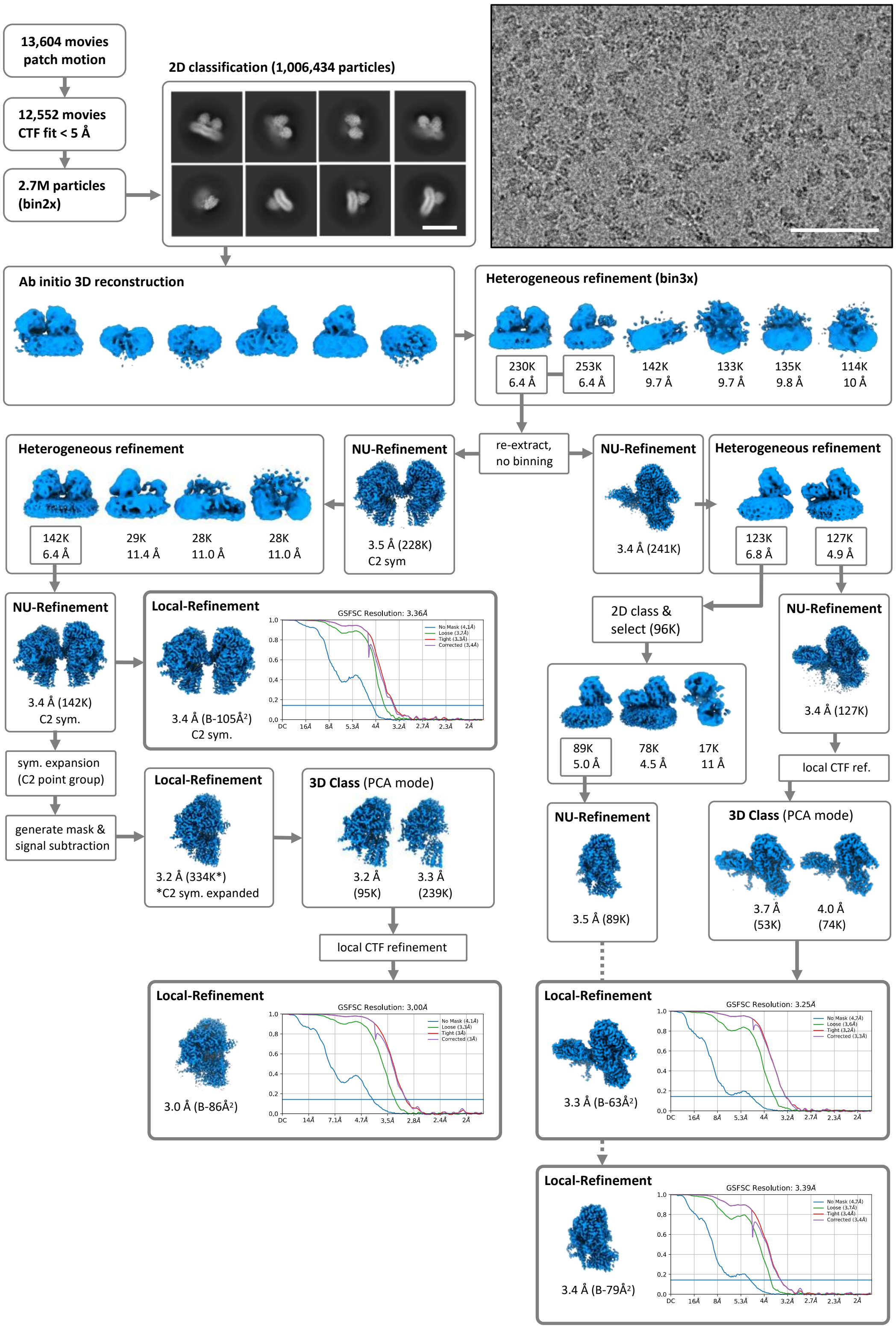

Sup-Fig 2: **Cryo-EM analysis of the SPT-Orm1 complexes**

Processing workflow for SPOT-dimer, SPOT-monomer and SPOTS complex. All processing steps were performed in cryoSPARC. Representative cryo-EM micrograph and 2D-class averages. 100 nm scale bar in micrograph and 20 nm scale bar in 2D class-averages.

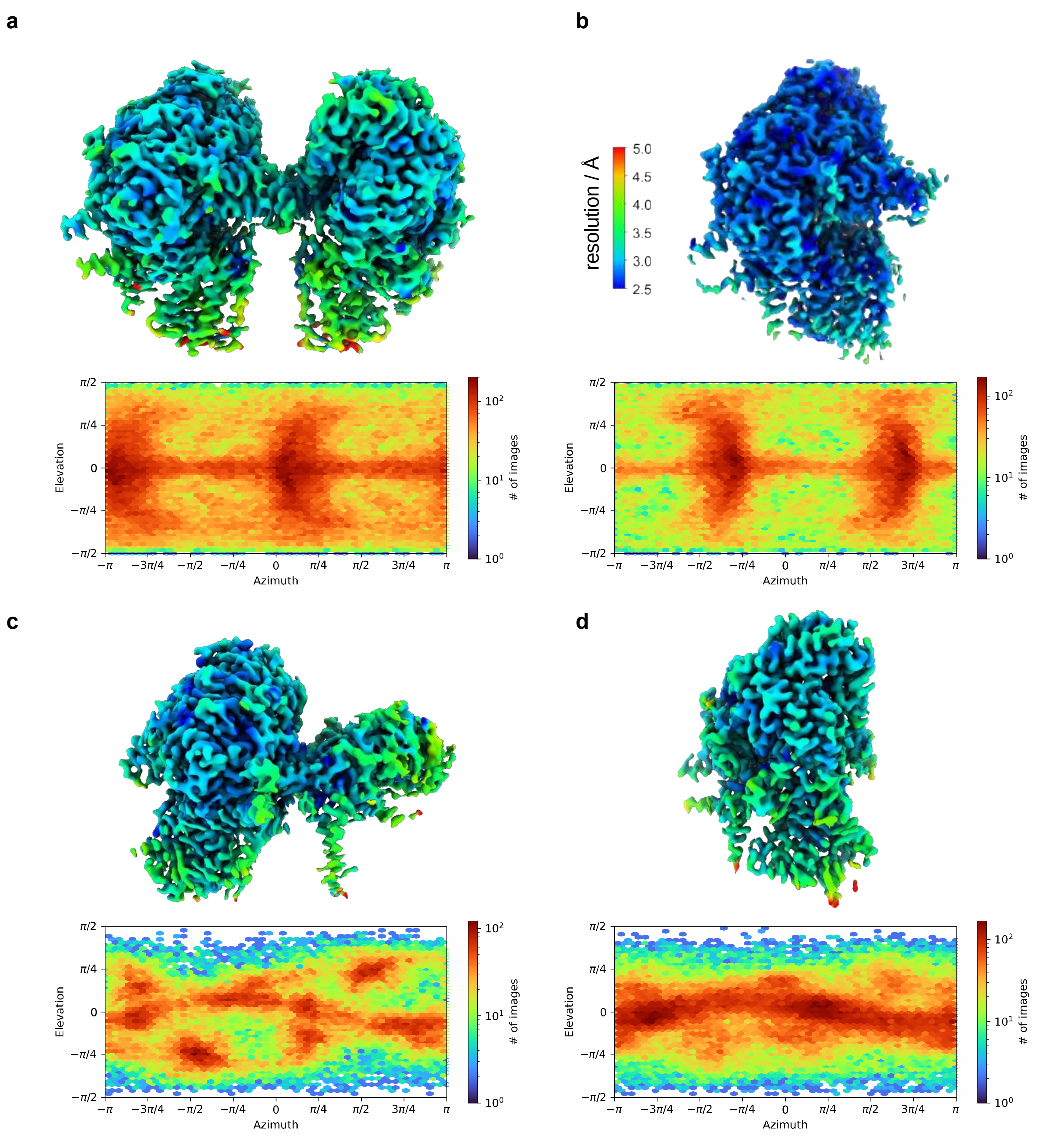

Sup-Fig 3: **Cryo-EM map evaluation**

Local resolution estimation and angular particle distribution of **a** SPOT-dimer (level 0.13), **b** masked SPOT-dimer (level 0.13), **c** SPOTS (level 0.18) and **d** SPOT-monomer (level 0.14).

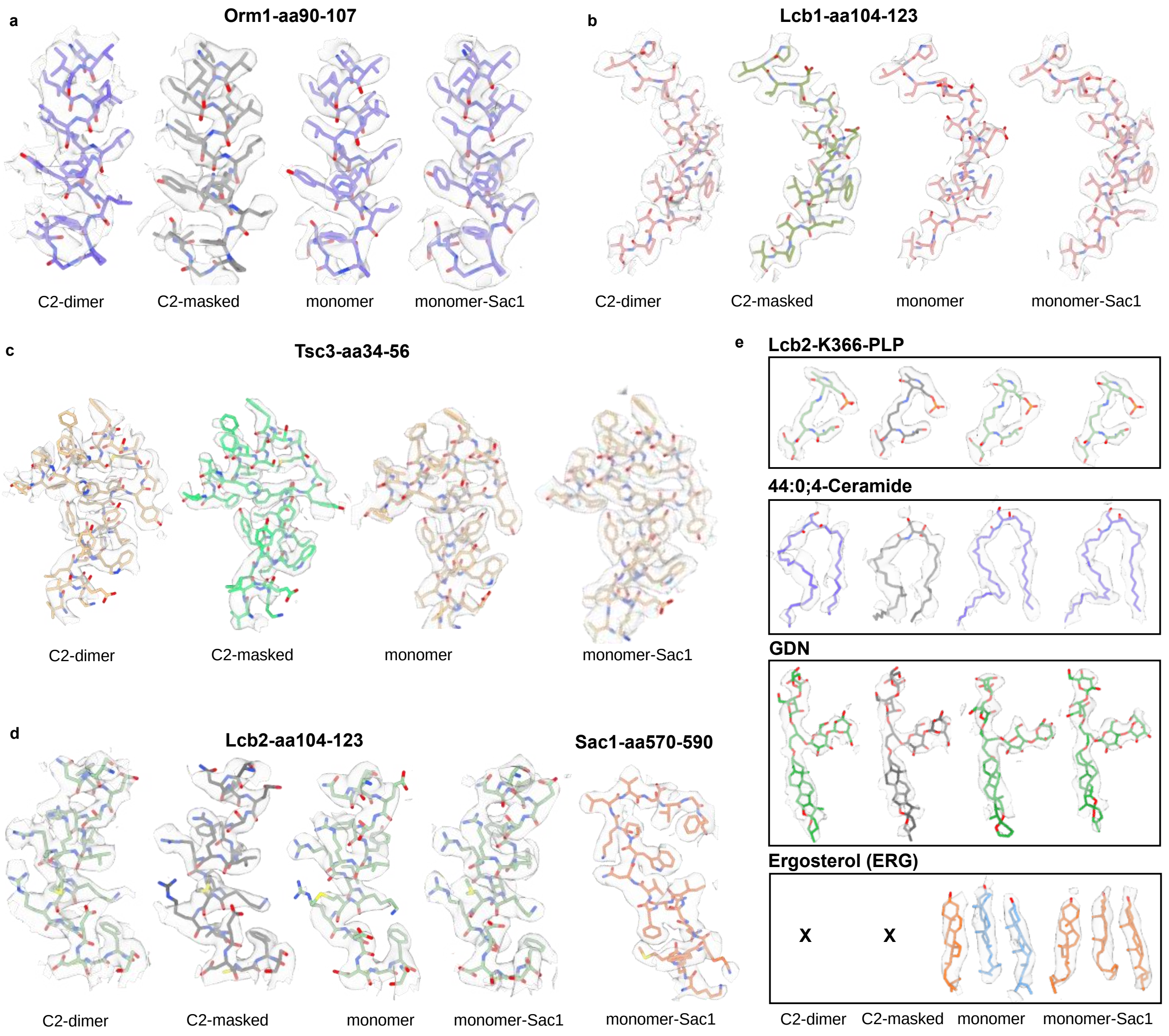

Sup-Fig 4: **Local cryo-EM density map quality of SPOT and SPOTS complexes**

Side-by-side comparison of selected residues and ligands within all cryo-EM density maps of **a** Orm1, **b** Lcb1, **c** Tsc3, **d** Lcb2, Sac1 and ligands in **e**. Ergosterol (ERG) was not present in maps denoted with an x. Contouring levels according to Supplementary Figure 3.

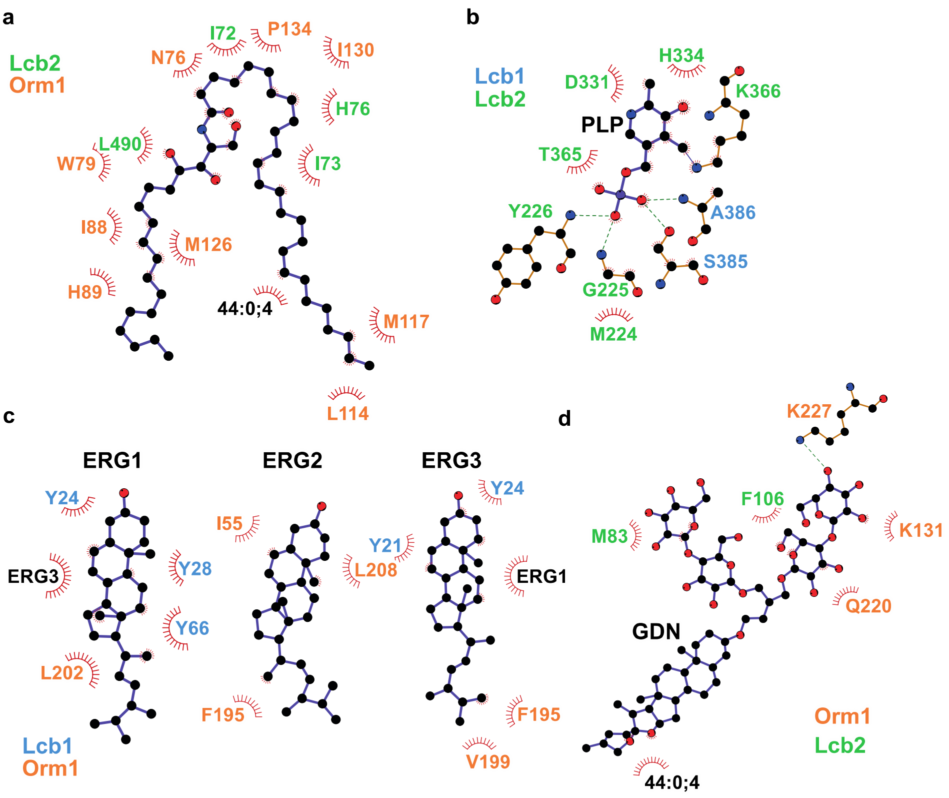

Sup-Fig 5: **Ligand interaction diagram of SPOT and SPOTS complexes**

2D ligand interaction diagram of ceramide 44:0;4 (**a**), internal aldimine between PLP and Lcb2^{K366} (**b),** ergosterols (ERG) (**c**) and glyco-diosgenin (GDN) (**d**). Key interacting residues are shown as ball and sticks with polar contacts given as green dotted lines. Diagrams were calculated and visualized with LigPlot+[1]. (a,b,d for all oligomers, c for SPOT-monomer and SPOTS).

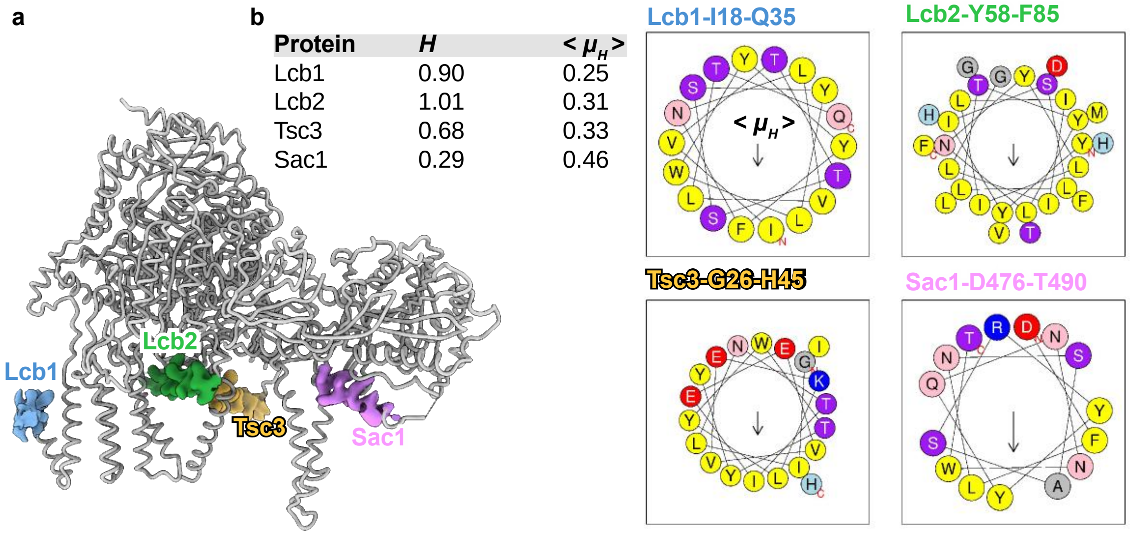

Protein	H	$<\mu_H>$
Lcb1	0.90	0.25
Lcb2	1.01	0.31
Tsc3	0.68	0.33
Sac1	0.29	0.46

Sup-Fig 6: **Amphipathic helix (AH) analysis of yeast Lcb1, Lcb2, Tsc3 and Sac1**

a Identification of four amphipathic helices in SPOTS involves Lcb1$^{I18\text{-}Q35}$, Lcb2$^{Y58\text{-}F85}$, Tsc3$^{G26\text{-}H45}$ and Sac1$^{D476\text{-}T490}$. **b** Physico-chemical properties, including the mean hydrophobic moment < μ_H > (arrow proportional, perpendicular to membrane plane) and hydrophobicity H were calculated with HeliQuest[2]. Helix wheels are colored according to their chemical polarity.

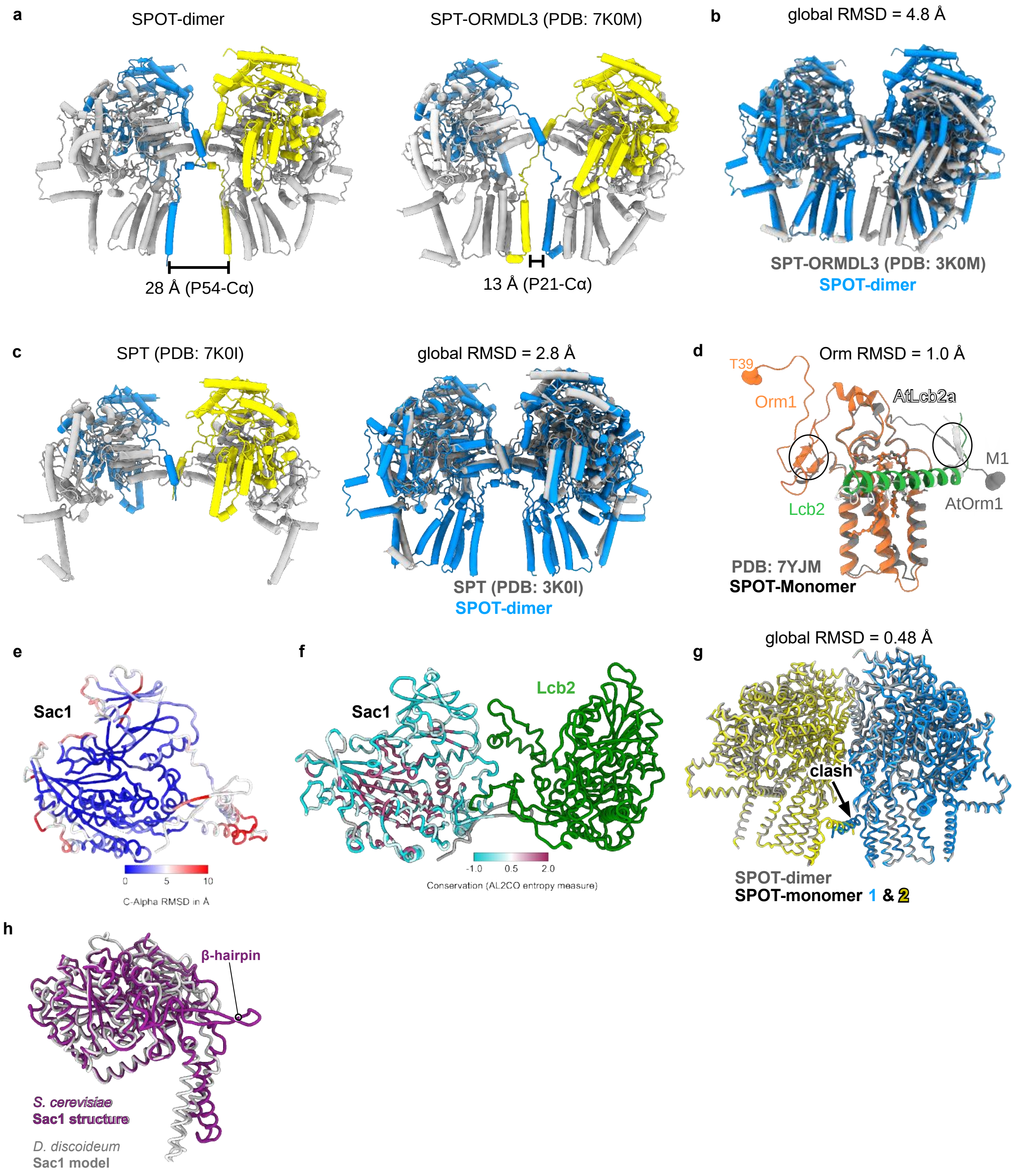

Sup-Fig 7: **Structural conservation within different SPOT oligomers**

a Side-by-side comparison of the Cα distances between the conserved proline residue in TM1 in the yeast SPOT-dimer and human SPT-ORMDL3 dimer (PDB: 7K0M[3]). Adjacent subunits are colored in blue and yellow for clarity. **b** Superposition of SPT-ORMDL3 and SPOT-dimer results in a global root-mean-square deviation (RMSD) of 4.8 Å. **c** Overview and superposition of ORMDL3-free human SPT (PDB: 7K0I[3]) and the SPOT-dimer results in a global RMSD of 2.8 Å. **d** Superposition of *Arabidopsis thaliana* (PDB: 7YJM[4], gray) and SPOT-monomer with a local Cα RMSD between the Orm1 subunits of 1.0 Å. Yeast Orm1 in orange and

Lcb2 in green. Orm1 N-termini depicted as spheres. **e** Cα RMSD between the Sac1 subunit from SPOTS and an AlphaFold prediction of *Dictyostelium discoideum* (Q55AW9). **f** Sequence conservation between yeast Sac1 and its homolog from *D. discoideum*. The Lcb2 subunit (green) is added for highlighting the interface. **g** Superposition of two SPOT-monomers (blue and yellow) onto the SPOT-dimer (grey). The sterical clash of Lcb1-TM0a is indicated with an arrow. **h** Superposition of an AlphaFold prediction of *D. discoideum* (Q55AW9, gray) and the structure of the SPOTS complex (violet).

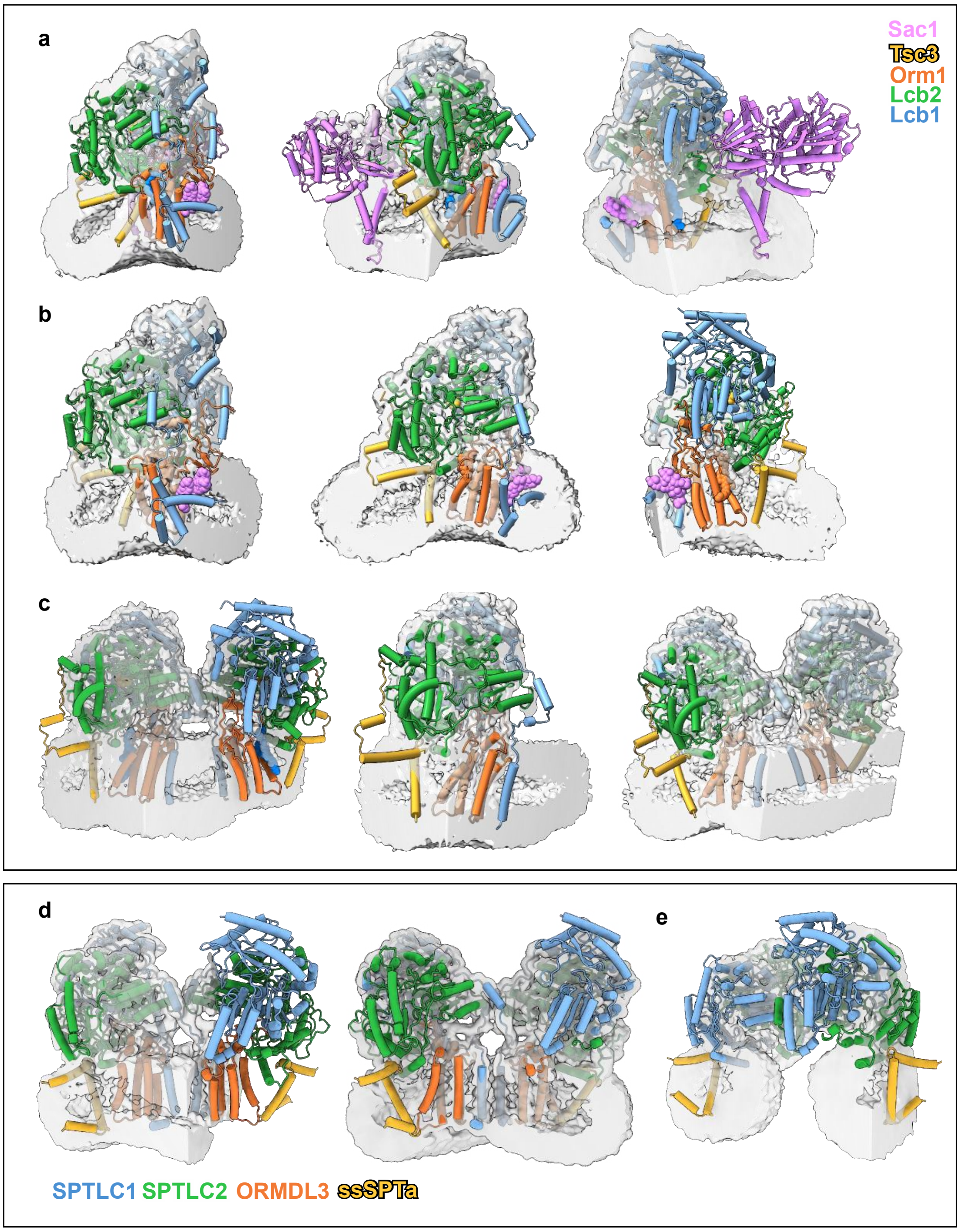

Sup-Fig 8: **Micelle curvature induction by SPT-ORMDL3 and yeast SPT oligomers**

Representative volume slices of **a** SPOTS **b** SPOT-monomer **c** SPOT-dimer **d** SPT-ORMDL3 (PDB: 7K0M, EMD-22602[3]) and **e** SPTLC1/SPLTC2/ssSPTa (PDB: 7K0I, EMD-22598[3]). EMDB maps were gaussian filtered by 1.5-fold standard deviations.

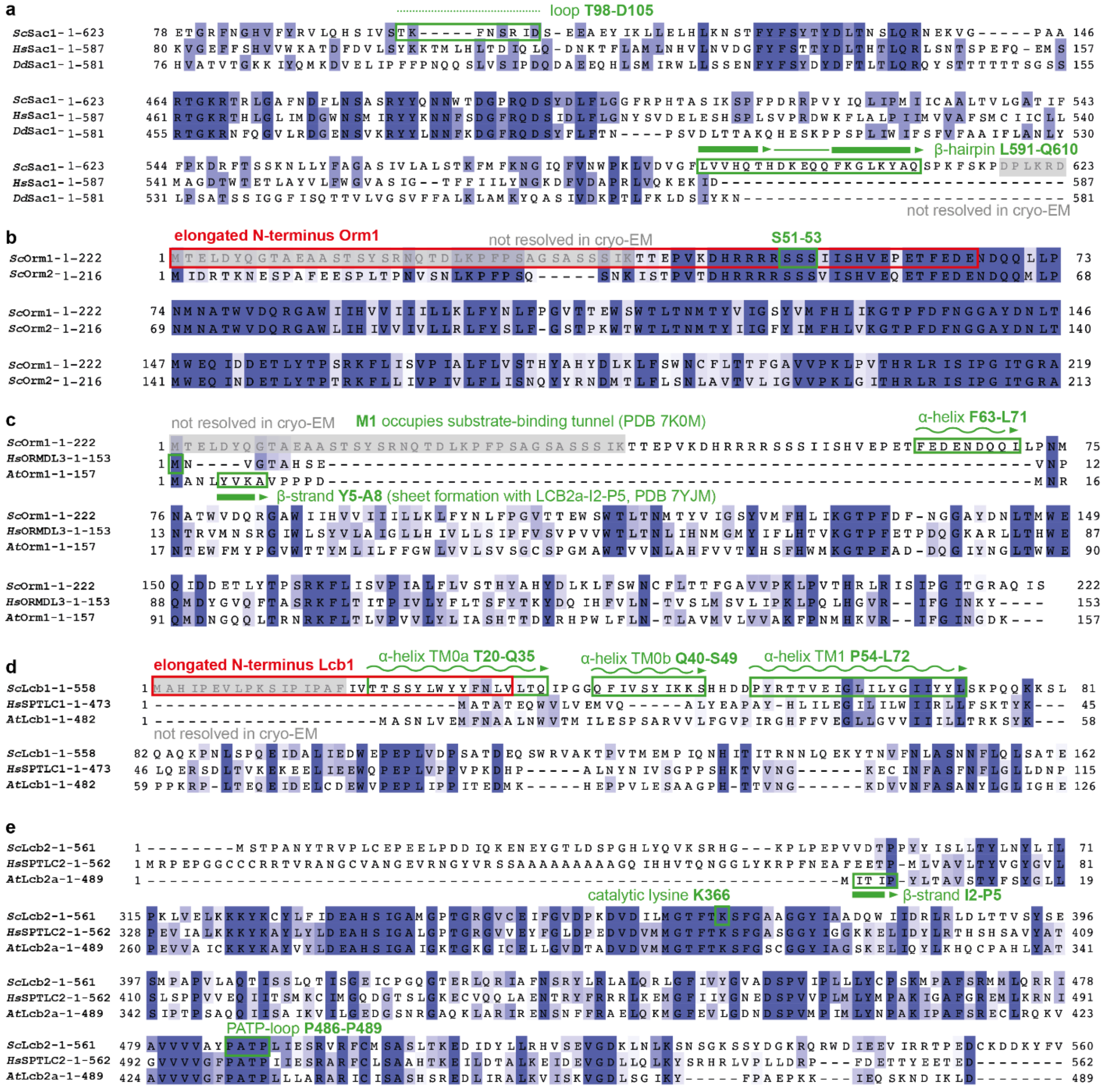

Sup-Fig 9: **Sequence conservation and unique features within different SPT complexes**

Clustal-based multiple-sequence alignment (MSA) of **a** Sac1 from *S. cerevisiae* (P32368), *H. sapiens* (Q9NTJ5) and *D. discoideum* (Q55AW9) with the C-terminal β-hairpin region and Lcb2 interacting loop T98-D105 highlighted in green. **b** Comparison of *S. cerevisiae* Orm1 (P53224) and Orm2 (Q06144). The elongated N-terminal region of Orm1 is colored in red and the mutated phosphorylation-sites S51-53 are marked in green. **c** Comparison of *H. sapiens* ORMDL3 (Q8N138), *A. thaliana* Orm1 (Q9C5I0) and yeast Orm1 (P53224). The N-terminal methionine is highlighted in green, which was observed to occupy the substrate binding tunnel in human SPT-ORMDL3 (PDB: 7K0M). *At*Orm1 Y5-A8 adopts a β-sheet with *At*Lcb2a I2-P5 (PDB: 7YJM); both highlighted in green. The Orm1 helix F63-L71 interacts with Lcb1/2 is highlighted in green. **d** Yeast Lcb1 (P25045) has an elongated N-terminal region, including helix TM0a (T20-Q35) and TM0b (Q40-S49), which are not present in human SPTLC1 (O15269). **e** Alignment of Lcb2

homologues from human SPTLC2 (O15270) and *Arabidopsis* Lcb2a (Q9LSZ9) with yeast Lcb2 (P40970). The PATP-loop and catalytic lysine are highlighted in green. Non-resolvable residues from cryo-EM are marked in light gray. Sequences are color coded by conservation with a cut-off at 30 %. MSAs were prepared with Jalview[5].

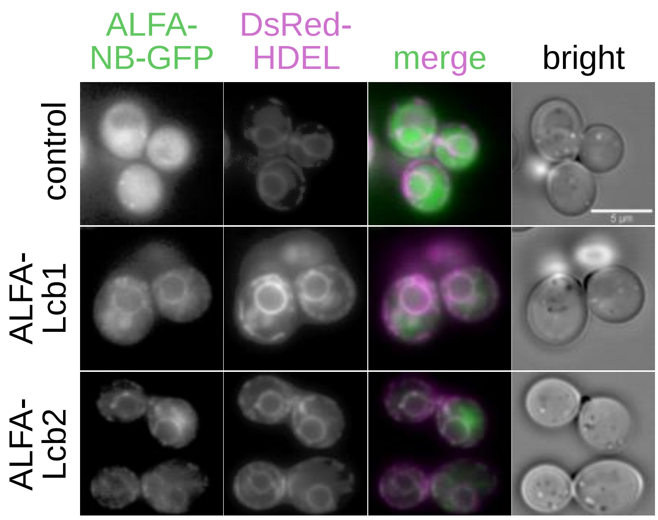

Sup-Fig. 10: **The N-terminus of Lcb1 faces the cytosol**

A cytosolic GFP-tagged ALFA nanobody (ALFA-NB-GFP) is expressed in control cells (upper panels), ALFA-Lcb1 cells (middle panels) and ALFA-Lcb2 cells (lower panels) also expressing *Ds*Red-HDEL. Both, ALFA-Lcb1 and ALFA-Lcb2 allow the recruitment of the otherwise cytosolic ALFA-NB-GFP to the ER membrane marked with *Ds*Red-HDEL showing that the N-termini of both proteins face the cytosol. GFP (left panels), *Ds*Red-HDEL (middle left panels), merged images (middle right panels) and brightfield images (right panels) are shown. Scale bar = 5 µm. Source data are provided as a Source Data file.

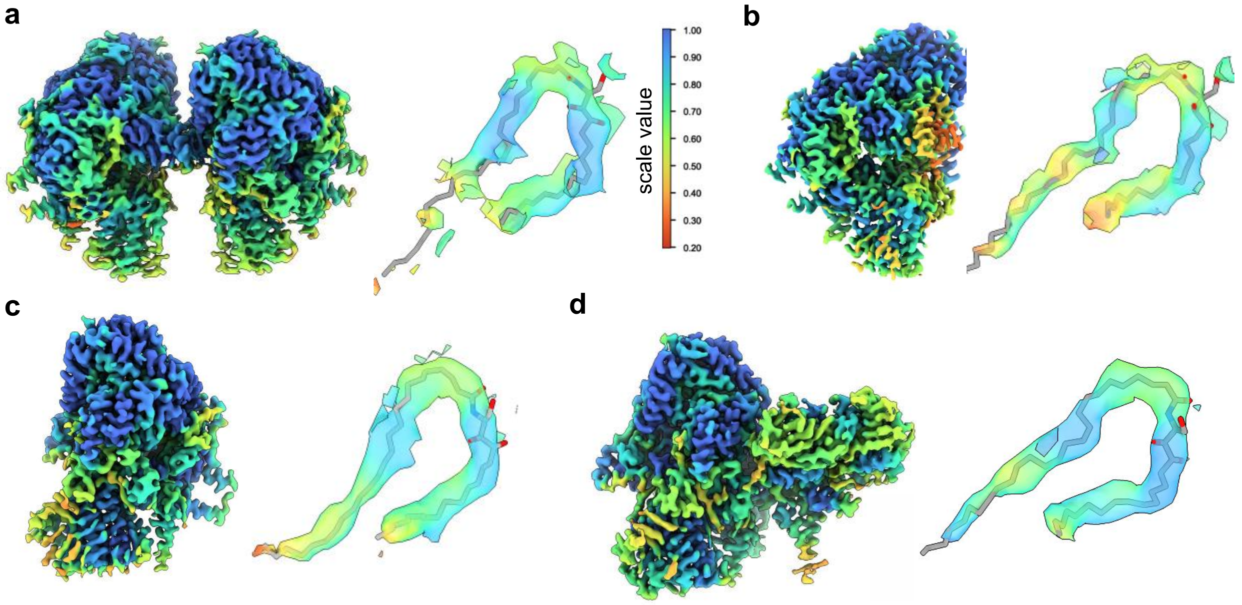

Sup-Fig. 11: **Estimation of compositional heterogeneity**

Scale-value color-coded cryo-EM densities for the overall reconstruction and local density for 44:0;4 ceramide are shown. **a** SPOT-dimer. **b** SPOT-dimer masked. **c** SPOT-monomer and **d** SPOTS-complex. All maps are contoured automatically by OccuPy[6]. Low compositional heterogeneity is shown in blue, high values are shown in red.

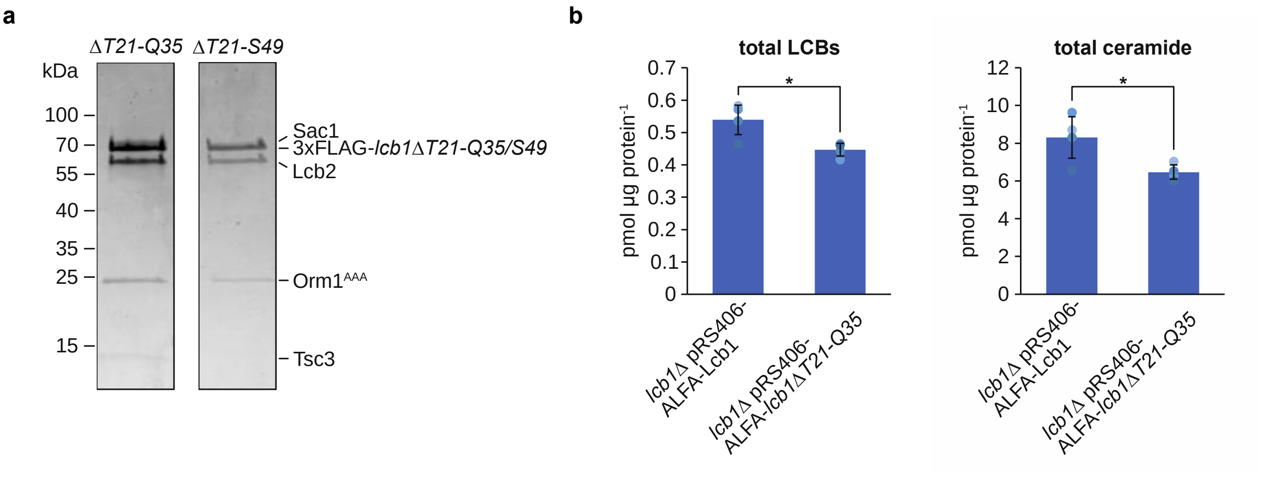

Sup-Fig. 12: **The TM0 helix affects SPT activity *in vivo* but not complex formation**

a Purification of SPOTS complex harboring the *lcb1ΔT21-Q35* (left) or *lcb1ΔT21-S49* mutants. SDS-PAGE gels after elution with FLAG peptide are shown. **b** Total LCB and ceramide levels were measured in cells expressing either the WT ALFA-tagged Lcb1 or the ALFA- *lcb1ΔT21-Q35* mutant. Data were analyzed using

a two-tailed t-test (*$p<0.05$) with n=4 biologically independent samples and data are presented as mean values ± SD. Exact *P* values are shown in Sup. Tab. 7. Source data are provided as a Source Data file.

Sup-Tab. 1: **Cryo-EM data collection, refinement and validation statistics**

	**SPOT-Dimer**	**SPOT-dimer masked**	**SPOTS**	**SPOT-monomer**
**Data Collection**				
Accession number	EMD-16469	EMD-16485	EMD-16468	EMD-16467
Magnification	130,000	130,000	130,000	130,000
Voltage / kV	200	200	200	200
Dose / e-Å-2	50	50	50	50
Pixel size / Å	0.924	0.924	0.924	0.924
Defocus range / µm	-2.0 to -0.8	-2.0 to -0.8	-2.0 to -0.8	-2.0 to -0.8
Recorded movies	13,604	13,604	13,604	13,604
Final particle images	141,900	94,884	53,236	89,484
Microscope	Glacios	Glacios	Glacios	Glacios
Camera	Falcon 4	Falcon 4	Falcon 4	Falcon 4
Energy Filter	Selectris	Selectris	Selectris	Selectris
**Image Processing**				
Initial model	AlphaFold 2	AlphaFold 2	AlphaFold 2	AlphaFold 2
Processing software	cryoSPARC (v.4)	cryoSPARC (v.4)	cryoSPARC (v.4)	cryoSPARC (v.4)
Symmetry imposed	C2	C2	C1	C1
Resolution (FSC0.143) / Å	3.4	3.0	3.3	3.4
Applied B-factor / Å2	-105	-86	-63	-79
**Model Refinement**				
PDB accession	8C82		8C81	8C80
Validation				
FSCmap-to-model (0.143) / Å	3.0		3.2	3.3
MolProbity score	1.68		1.45	1.33
Clash Score	7.93		3.98	3.71
Composition				
Atoms	21,208		15,938	10,968
Protein residues	2,634		1,966	1,350
Ligands	6		6	6
Bonds (R.M.S.D.)				
Length (Å)	0.003		0.003	0.003
Angles (°)	0.653		0.541	0.615
B-factors (min/max/mean)				
Protein residues	50.62/143.88/63.21		60.65/221.62/100.09	54.22/176.06/87.84
Ligand	23.35/152.04/114.63		83.24/117.15/105.19	90.15/120.51/106.88
Ramachandran plot (%)				
Favored	96.33		96.06	97.02
Allowed	3.67		3.94	2.98
Outliers	0.00		0.00	0.00
Rotamer outliers (%)	0.00		0.00	0.00

Sup-Tab. 2: **List of all yeast strains used in this study**

Strain	Genotype	Reference
FFY3085	MATα *leu2-3,112 trp1-1 can1-100 ura3-1 ade2-1 his3-11,15 lcb1Δ::hphNT1 pRS415-3xFLAG-LCB1::LEU2*	This study
FFY4354	MATα *leu2-3,112 trp1-1 can1-100 ura3-1 ade2-1 his3-11,15 lcb1Δ::hphNT1 pRS415-3xFLAG-LCB1::LEU2 sac1Δ1-264::kanMX6 pRS403-ALFA-SAC1::HIS3*	This study
FFY4192	MATa/MATα *leu2-3,112 trp1-1 can1-100 ura3-1 ade2-1 his3-11,15 orm1Δ::natNT2 orm2Δ::hphNT1 pRS404-GAL1-TSC3::TRP1 pRS406-GAL1-ORM1^{AAA}::URA3 pRS406-GAL1-3xFLAG-LCB1::URA3 pRS405-GAL1-LCB2::LEU2 pRS403-GAL1-SAC1::HIS3*	This study
FFY4133	MATa/MATα *leu2-3,112 trp1-1 can1-100 ura3-1 ade2-1 his3-11,15 orm1Δ::natNT2 orm2Δ::hphNT1 pRS404-GAL1-TSC3::TRP1 pRS406-GAL1-3xFLAG-LCB1::URA3 pRS405-GAL1-LCB2::LEU2 pRS403-GAL1-SAC1::HIS3*	This study
FFY5161	MATα *leu2-3,112 ura3-52 his3-Δ200 trp1-Δ901 ADE2 suc2-Δ9 GAL lys2-801 lcb2Δ::hphNT1 pRS403-ALFA-LCB2::HIS3 YIp128-ALFAnb-GFP::LEU2 pRS404-DsRed-HDEL::TRP1*	This study
FFY5162	MATα *leu2-3,112 ura3-52 his3-Δ200 trp1-Δ901 ADE2 suc2-Δ9 GAL lys2-801 lcb1Δ::hphNT1 pRS406-ALFA-LCB1::URA3 YIp128-ALFAnb-GFP::LEU2 pRS404-DsRed-HDEL::TRP1*	This study
FFY5228	MATα *leu2-3,112 ura3-52 his3-Δ200 trp1-Δ901 ADE2 suc2-Δ9 GAL lys2-801 YIp128-ALFAnb-GFP::LEU2 pRS404-DsRed-HDEL::TRP1*	This study
FFY5326	MATa/MATα *leu2-3,112 trp1-1 can1-100 ura3-1 ade2-1 his3-11,15 lcb2Δ::kanMX6/LCB2 pRS405-ALFA-LCB2^{Y485S}::LEU2*	This study
FFY5322	MATa/MATα *leu2-3,112 trp1-1 can1-100 ura3-1 ade2-1 his3-11,15 lcb2Δ::kanMX6/LCB2 pRS405-ALFA-LCB2^{Y110S}::LEU2*	This study
FFY5472	*MATα leu2-3,112 trp1-1 can1-100 ura3-1 ade2-1 his3-11,15 lcb2Δ::kanMX6 pRS405-ALFA-LCB2::LEU2*	This study
FFY5478	*MATα leu2-3,112 trp1-1 can1-100 ura3-1 ade2-1 his3-11,15 lcb2Δ::kanMX6 pRS405-ALFA-LCB2^{Y485S}::LEU2*	This study
FFY5459	*MATα leu2-3,112 trp1-1 can1-100 ura3-1 ade2-1 his3-11,15 pRS405-ALFA-LCB2^{Y110S}::LEU2*	This study
FFY5564	*MATa leu2-3,112 trp1-1 can1-100 ura3-1 ade2-1 his3-11,15 lcb2Δ::kanMX6 pRS403-ALFA-LCB2::HIS3 orm1Δ::natNT2 pRS406-ALFA-ORM1::URA3*	This study
FFY5552	*MATα leu2-3,112 trp1-1 can1-100 ura3-1 ade2-1 his3-11,15 lcb2Δ::kanMX6 pRS403-ALFA-LCB2^{L69F}::HIS3 orm1Δ::natNT2 pRS406-ALFA-ORM1^{M126F}::URA3*	This study
FFY5553	*MATα leu2-3,112 trp1-1 can1-100 ura3-1 ade2-1 his3-11,15 lcb2Δ::kanMX6 pRS403-ALFA-LCB2^{L69F}::HIS3 orm1Δ::natNT2 pRS406-ALFA-ORM1^{G122F}::URA3*	This study
FFY4324	*MATα leu2-3,112 trp1-1 can1-100 ura3-1 ade2-1 his3-11,15 sac1Δ1-264::kanMX6*	This study
FFY5140	*MATa leu2-3,112 trp1-1 can1-100 ura3-1 ade2-1 his3-11,15 sac1Δ1-264::kanMX6 pRS403-ALFA-SAC1::HIS3*	This study
FFY5147	*MATα leu2-3,112 trp1-1 can1-100 ura3-1 ade2-1 his3-11,15 sac1Δ1-264::kanMX6 pRS403-ALFA-sac1Δ574-623::HIS3*	This study
FFY5462	*MATα leu2-3,112 trp1-1 can1-100 ura3-1 ade2-1 his3-11,15 pRS403::HIS3*	This study
FFY5495	*MATa/MATα leu2-3,112 trp1-1 can1-100 ura3-1 ade2-1 his3-11,15 lcb1Δ::hphNT1/lcb1Δ::hphNT1 pRS406-ALFA-LCB1::Ura3 pRS403-3xFLAG-LCB1::His3*	This study

FFY5509	*MATa/MATα leu2-3,112 trp1-1 can1-100 ura3-1 ade2-1 his3-11,15 orm1Δ::natNT2/orm1Δ::natNT2 orm2Δ::hphNT1/orm2Δ::hphNT1 pRS404-GAL1-TSC3::TRP1 pRS406-GAL1-ORM1*^{AAA}*::URA3 pRS406-GAL1-3xFLAG-lcb1ΔT21-Q35::URA3 pRS405-GAL1-LCB2::LEU2 pRS403-GAL1-SAC1::HIS3*	This study
FFY5510	*MATa/MATα leu2-3,112 trp1-1 can1-100 ura3-1 ade2-1 his3-11,15 orm1Δ::natNT2/orm1Δ::natNT2 orm2Δ::hphNT1/orm2Δ::hphNT1 pRS404-GAL1-TSC3::TRP1 pRS406-GAL1-ORM1*^{AAA}*::URA3 pRS406-GAL1-3xFLAG-lcb1ΔT21-S49::URA3 pRS405-GAL1-LCB2::LEU2 pRS403-GAL1-SAC1::HIS3*	This study
FFY5449	*MATα leu2-3,112 trp1-1 can1-100 ura3-1 ade2-1 his3-11,15 lcb1Δ::hphNT1 pRS406-ALFA-LCB1::URA3*	This study
FFY5439	*MATα leu2-3,112 trp1-1 can1-100 ura3-1 ade2-1 his3-11,15 lcb1Δ::hphNT1 pRS406-ALFA-lcb1ΔT21-Q35::URA3*	This study

Sup-Tab. 3: **List of all *Dictyostelium discoideum* lines used in this study**

**Strain**	**Plasmid used for transformation**	**Reference**
GFP-Sac1	pDM317-GFP-Sac1	Barisch lab (Osnabrück)
GFP	pDM317-GFP	Barisch lab (Osnabrück)

Sup-Tab. 4: **List of all plasmids used in this study**

**Plasmid**	**Reference**
pRS415-3xFLAG-*LCB1*::*LEU2*	Schmidt lab (Innsbruck)
pRS403-ALFA-*SAC1*::*HIS3*	This study
pRS406-GAL1-*ORM1*^{AAA}::*URA3*	This study
pRS404-GAL1-*TSC3*::*TRP1*	This study
pRS403-GAL1-*SAC1*::*HIS3*	This study
pRS405-GAL1-*LCB2*::*LEU2*	This study
pRS406-GAL1-3xFLAG-*LCB1*::*URA3*	This study
pRS404-DsRed-HDEL::*TRP1*	This study
YIp128-ALFA-NB-GFP::*LEU2*	Heinisch lab (Osnabrück)
pRS403-ALFA-*LCB2*::*HIS3*	This study
pRS406-ALFA-*LCB1*::*URA3*	This study
pDM317-GFP-*SAC1* (G418)	Vormittag *et al.*, 2023[7]
pDM317-GFP (G418)	Vormittag *et al.*, 2023[7]
pRS405-ALFA-*LCB2*^{Y485S}::*LEU2*	This study
pRS405-ALFA-*LCB2*^{Y110S}::*LEU2*	This study
pRS405-ALFA-*LCB2*::*LEU2*	This study
pRS406-ALFA-*ORM1*::*URA3*	This study
pRS403-ALFA-*LCB2*^{L69F}::*HIS3*	This study
pRS406-ALFA-ORM1^{M126F}::*URA3*	This study
pRS406-ALFA-*ORM1*^{G122F}::*URA3*	This study
pRS403-ALFA-*sac1Δ574-623*::*HIS3*	This study
pRS403::*HIS3*	Sikorski and Hieter, 1989[8]

pRS403-3xFLAG-*LCB1*::*HIS3*	This study
pRS406-GAL1-3xFLAG-*lcb1ΔT21-Q35*::*URA3*	This study
pRS406-GAL1-3xFLAG-*lcb1ΔT21-S49*::*URA3*	This study
pRS406-ALFA-*lcb1ΔT21-Q35*::*URA3*	This study

Sup-Tab 5: **List of all oligo nucleotides used in this study**

**Name**	**Sequence**
ALFA tag rev	TGG TTC GGT TAA TCT TCT TC
Sac1_aa2_for	ACA GGT CCA ATA GTG TAC
pRS406_for_Orm1	ATGACCGAATTAGATTATCAAGGAACTG
pRS406_rev_Orm1	GGCCCTACGCGCTCTAGA
GAL1_for_OH_Orm1 promoter	CTAGAGCGCGTAGGGCCAGTACGGATTAGAAGCCGC
GAL1_rev_OH_ORM 1	TAATCTAATTCGGTCATGTTTTTTCTCCTTGACGTTAAAG
pRS404_GAL1_for	TATATCTAGAACTAGTGGATCCCC
pRS404_GAL1_rev	GTTTTTTCTCCTTGACGTTAAAG
TSC3_OH_GAL1pr_f or	CGTCAAGGAGAAAAAACATGACACAACATAAAAGCTCG
TSC3_OH_pRS404_ rev	GGGGATCCACTAGTTCTAGATATATCTGTGACTCGGATATGGAG
pRS403_Sac1_for	ATGACAGGTCCAATAGTGTAC
pRS403_Sac1pr_rev	CGATCAGGACGTCAGGG
GAL1_OH_Sac1pr_f or	CCCTGACGTCCTGATCGAGTACGGATTAGAAGCCG
GAL1_OH_Sac1_rev	CACTATTGGACCTGTCATGTTTTTTCTCCTTGACGTTAAAG
pRS405_Lcb2_for	ATGAGTACTCCTGCAAACTATACC
pRS405_Lcb2_rev	CACCCAATCACCGCGCTT
GAL1pr_OH_Lcb2pr _for	AAGCGCGGTGATTGGGTGAGTACGGATTAGAAGCCG
GAL1pr_OH_Lcb2_r ev	TTTGCAGGAGTACTCATGTTTTTTCTCCTTGACGTTAAAG
pRS406_FLAG_LCB 1_for	ATGGCACACATCCCAGAG
pRS406_FLAG_LCB 1_rev	GACAGAGCAGTATGTGAGG
GAL1pr_OH_Lcb1pr _for	CCTCACATACTGCTCTGTCAGTACGGATTAGAAGCCG
GAL1pr_OH_Lcb1pr _rev	TCTGGGATGTGTGCCATGTTTTTTCTCCTTGACGTTAAAG
SAC1_KO_Rev	CAGCCCAGTATATTGGCACAGATCCTCTTGTCTGTAAGAAGGAGA TCGATGAATTCGAGCTCG
SAC1_S1 new	ATAATATTTATATACACGTATATTTTCTCGTCTAGATATGcgtacgctgc aggtcgac
Lcb1_S1	GTT ATT TAT CCT TTT TTC TTC CTT CCC ACC CAA AAA AAA AAA GCA ATG CGT ACG CTG CAG GTC GAC
LCB1 S2	ATA TAT ATG TGC GTG TGC ATA TAC TGG CTT TCT ATT TTT AAT CGA TGA ATT CGA GCT CG
Orm1_S2	AAA ATA TAA ATA TAG CAA AAA CAT CTA GAT ACA AGA TTG AAA TAA ACT ATG TTC AAT CGA TGA ATT CGA GCT CG
ORM1_S1	AAG CAG AGT TAT TCT TAT TTT GTA TTT CAT TGC ATT TTT ATC CAT TTA GTT AAT GCG TAC GCT GCA GGT CGA C
ORM2_S1	GAA TTA ACG CAA GAC TAT ACC ATT ATA AAA ACG CAT AAG AAA CAG TTT CAT CAT GCG TAC GCT GCA GGT CGA C
Orm2_S2	Orm2 S2

Lcb2_S1	AAGATTCCACACACTTTATTGTGATAGTTTTCAAAGTAAAAAGTAA TAGATTATGCGTACGCTGCAGGTCGAC
Lcb2_S2	ACGTCTTCCAGAAATTTTGTAATTTTTCACCTAACTAGCAATTAGG TAAAATTCAATCGATGAATTCGAGCTCG
backbone_exchange _vec_for	GGCGTAATCATGGTCATAGC
backbone_exchange _vec_rev	CTCACTGGCCGTCGTTTTAC
backbone_exchange _ins_for	GTAAAACGACGGCCAGTGAG
backbone_exchange _ins_rev	GCTATGACCATGATTACGCC
pALFA-Lcb1-Q5-for	TTACGTCGTCGTTTGACCGAACCCAAATCAATACCGATTCCGG
pALFA-Lcb1-Q5-rev	TTCCTCTTCCAACCTGGAGGGGGGTAAAACCTCTGGGATG
Q5_pALFA_Lcb2_for	TTACGTCGTCGTTTGACCGAACCCATGAGTACTCCTGCAAAC
Q5_pALFA_Lcb2_rev	TTCCTCTTCCAACCTGGAGGGCATAATCTATTACTTTTTACTTTGA AAAC
Lcb1_I36_for	ATCCCGGGAGGCCAATTC
Lcb1_T20_rev	GGTAACAATAAATGCCGGAATCGG
Lcb1_H50_for	CATCATGACGATCCATACAGGACC
Lcb2_Y110S_Q5_rev	AAATTTGAAAACCAAGGTGC
Lcb2_Y110S_Q5_for	CGAGAGTTTTTCTGTCAGGAGAATTAAAATG
Lcb2_Y485S_Q5_for	TGTTGTTGCTTCTCCTGCTACTC
Lcb2_Y485S_Q5_rev	ACAACAGCAATCCGTCTTTG
Orm1_G122F_for	ATA TGT TAT TTT TTC CTA TGT CAT GTT CCA TCT G
Orm1_G122F_rev	GTC ATA TTA GTC AGA GTC C
Orm1_M126F_for	GTC CTA TGT CTT TTT CCA TCT GAT TAA G
Orm1_M126F_rev	CCA ATA ACA TAT GTC ATA TTA GTC
Lcb2_L69F_for	TCT AAA TTA TTT TAT TCT GAT TAT ATT AGG TC
Lcb2_L69F_rev	TAT GTT AAC AAA GAA ATG TAA TAA G

Sup-Tab. 6: **List of used transitions for targeted lipidomics**

**Description**	**Q1 mass**	**Q2 mass**	**CE (V)**	**CXP (V)**	**DP (V)**	**EP (V)**
Ceramide d17:1/24:0	636.629	249.8	47	13	120	10
Phytoceramide 42:0;3	668.655	282.4	50	5	100	5
Phytoceramide 42:0;4	684.650	282.4	50	5	100	5
Phytoceramide 42:0;5	700.645	282.4	50	5	100	5
Phytoceramide 44:0;3	696.686	282.4	50	5	100	5
Phytoceramide 44:0;4	712.681	282.4	50	5	100	5
Phytoceramide 44:0;5	728.676	282.4	50	5	100	5
Sphingosine d17:1	286.274	69	55	10	51	10
3-ketosphinganine 18:0	300.290	60	25	6	156	10
Phytosphingosine 18:0	318.300	60	45	10	166	10
Phytosphingosine 20:0	346.332	60	45	10	166	10
Dihydrosphingosine 18:0	302.305	60	23	8	66	10
Dihydrosphingosine 20:0	330.337	60	23	8	66	10

Sup-Tab. 7: **List of all exact *P*-values of LCB and ceramide analyses**

**Measure**		***P*-value**
3-KS 18:0		
	ALFA-Lcb2^{Y485S}	0.02044
	ALFA-Lcb2^{Y110S}	0.25469
	ALFA-Lcb2^{L69F} ALFA-Orm1^{M126F}	0.00837
	ALFA-Lcb2^{L69F} ALFA-Orm1^{G122F}	0.00492
	sac1Δ	$3.37 \cdot 10^{-8}$
	sac1Δ574	0.7706
Total LCBs 18:0		
	ALFA-Lcb2^{Y485S}	$1.62 \cdot 10^{-5}$
	ALFA-Lcb2^{Y110S}	$1.05 \cdot 10^{-5}$
	ALFA-Lcb2^{L69F} ALFA-Orm1^{M126F}	$2.89 \cdot 10^{-4}$
	ALFA-Lcb2^{L69F} ALFA-Orm1^{G122F}	0.00101
	sac1Δ	$1.53 \cdot 10^{-9}$
	sac1Δ574	$1.50 \cdot 10^{-5}$
Total LCBs	ALFA-*lcb1ΔT21-Q35*	0.018446273
Total ceramides		
	ALFA-Lcb2^{Y485S}	0.00403
	ALFA-Lcb2^{Y110S}	0.0015
	ALFA-Lcb2^{L69F} ALFA-Orm1^{M126F}	0.01848
	ALFA-Lcb2^{L69F} ALFA-Orm1^{G122F}	0.01669
	sac1Δ	0.00422
	sac1Δ574	$3.41 \cdot 10^{-4}$
	ALFA-*lcb1ΔT21-Q35*	0.034623781

Supplementary References

1. Laskowski, R. A. & Swindells, M. B. LigPlot+: Multiple ligand-protein interaction diagrams for drug discovery. *J Chem Inf Model* **51**, 2778–2786 (2011).

2. Gautier, R., Douguet, D., Antonny, B. & Drin, G. HELIQUEST: a web server to screen sequences with specific alpha-helical properties. *Bioinformatics* **24**, 2101–2102 (2008).

3. Wang, Y. *et al.* Structural insights into the regulation of human serine palmitoyltransferase complexes. *Nature Structural & Molecular Biology 2021 28:3* **28**, 240–248 (2021).

4. Liu, P. *et al.* Mechanism of sphingolipid homeostasis revealed by structural analysis of Arabidopsis SPT-ORM1 complex. *Sci Adv* **9**, (2023).

5. Troshin, P. V. *et al.* JABAWS 2.2 distributed web services for Bioinformatics: protein disorder, conservation and RNA secondary structure. *Bioinformatics* **34**, 1939–1940 (2018).

6. Forsberg, B. O., Shah, P. N. M. & Burt, A. A robust normalized local filter to estimate compositional heterogeneity directly from cryo-EM maps. *bioRxiv* 2023.01.18.524529 (2023) doi:10.1101/2023.01.18.524529.

7. Vormittag, S. *et al.* Legionella- and host-driven lipid flux at LCV-ER membrane contact sites promotes vacuole remodeling. *EMBO Rep* (2023) doi:10.15252/EMBR.202256007.

8. Sikorski, R. S. & Hieter, P. A system of shuttle vectors and yeast host strains designed for efficient manipulation of DNA in Saccharomyces cerevisiae. *Genetics* **122**, 19–27 (1989).
