# Supplementary material for: Structure of the ceramide-bound SPOTS complex
Source: Nat Commun. 2023 Oct 4;14:6196. doi: 10.1038/s41467-023-41747-z (PMC10550967; doi:10.1038/s41467-023-41747-z)
Supplement: Supplementary file 3 — Description of additional supplementary data [file 41467_2023_41747_MOESM3_ESM.docx]

**Description of Additional Supplementary Data**

**File name:** Supplementary Data 1

**Description:** Results of analyzed mass spectrometry data using MaxQuant or PASer for pulldown experiments and purified proteins.
